# Transforming self-reported outcomes from a stroke register to the modified Rankin Scale: a cross-sectional, explorative study

**DOI:** 10.1038/s41598-020-73082-4

**Published:** 2020-10-14

**Authors:** Tamar Abzhandadze, Malin Reinholdsson, Annie Palstam, Marie Eriksson, Katharina S. Sunnerhagen

**Affiliations:** 1 Institute of Neuroscience and Physiology, Rehabilitation Medicine, University of Gothenburg, Per Dubbsgatan 14, fl. 3, 413 45 Gothenburg, Sweden; 2 Department of Occupational Therapy and Physiotherapy, Sahlgrenska University Hospital, Gothenburg, Sweden; 3 Department of Statistics, USBE, Umeå University, Umeå, Sweden

**Keywords:** Health care, Neurology, Signs and symptoms

## Abstract

The aim was to create an algorithm to transform self-reported outcomes from a stroke register to the modified Rankin Scale (mRS). Two stroke registers were used: the Väststroke, a local register in Gothenburg, Sweden, and the Riksstroke, a Swedish national register. The reference variable, mRS (from Väststroke), was mapped with seven self-reported questions from Riksstroke. The transformation algorithm was created as a result of manual mapping performed by healthcare professionals. A supervised machine learning method—decision tree—was used to further evaluate the transformation algorithm. Of 1145 patients, 54% were male, the mean age was 71 y. The mRS grades 0, 1 and 2 could not be distinguished as a result of manual mapping or by using the decision tree analysis. Thus, these grades were merged. With manual mapping, 78% of the patients were correctly classified, and the level of agreement was almost perfect, weighted Kappa (K_w_) was 0.81. With the decision tree, 80% of the patients were correctly classified, and substantial agreement was achieved, K_w_ = 0.67. The self-reported outcomes from a stroke register can be transformed to the mRS. A mRS algorithm based on manual mapping might be useful for researchers using self-reported questionnaire data.

## Introduction

Stroke registers are valuable data sources for scientific studies and for understanding the consequences of stroke^1,2^. For stroke quality registers, data are gathered by staff or reported by patients. Self-reported information might have validity and reliability issues, but incorporating standardized assessment tools into quality registers can lead to an increased number of questions that might be difficult for the patients to answer.

The modified Rankin Scale (mRS) is one of the frequently used assessment instruments in stroke-related studies^3–7^, but the administration of this instrument is staff dependent; the assessments are to be performed by trained personnel, either in person or by telephone interviews^8^. Therefore, it can be difficult to obtain information from large geographical areas and at several time points. Eriksson et al.^9^ transformed five self-reported outcomes from the Swedish national stroke register, Riksstroke, to the mRS. The manual mapping method was used. Eriksson et al.'s^9^ algorithm allowed Riksstroke-based research to be compared across studies in which the mRS was used^10^. Since the first transformation algorithm was developed, the questions in Riksstroke have been changed. Moreover, the previous algorithm could not distinguish mRS grades 0, 1 and 2 from each other^9^. The supervised machine learning method could potentially be used to transform the self-reported data with more accuracy. The machine learning algorithms help label the input data and group the output into several classes.

The aims of this study were to create a new transformation algorithm for the modified Rankin Scale-Riksstroke (mRS-RS) based on self-reported outcomes from Riksstroke and to distinguish mRS-RS grades 0, 1 and 2 from each other. These aims were addressed by using a combination of manual mapping and a supervised machine learning method.

## Methods

### Study sample and procedure

This is a cross-sectional, explorative, register-based study, part of the Physical Activity Pre-Stroke In GOThenburg project^11^. Two quality registers were used: the Väststroke, a local stroke register in Gothenburg, Sweden, and the Riksstroke, a Swedish national register for stroke. Acute care and three-month follow-up data recorded from January 1, 2015 to August 31, 2018 were extracted. Data for acute care were registered by hospital staff. The 3-month follow-up data were collected using postal, self-administered questionnaires as well as telephone interviews by trained nurses. The Väststroke and Riksstroke registers were linked by a statistician at Riksstroke by using the patients’ unique personal identification numbers.

The inclusion criteria applied in this study were as follows: patients with first-ever stroke, diagnosed according to the International Classification of Diseases codes (ICD -10), those with an age > 18 y, complete data on those patients with mRS data registered in the Väststroke register and responses to Riksstroke's questions that were to be used in the algorithm, as shown in Supplementary Table S1.

### Ethics and informed consent

The data file that was used in the study was anonymized and individual patients could not be identified. The study was approved by the regional ethical review board in Gothenburg (# 346 – 16) and the Swedish Ethical Review Authority (the amendment # 2019-01251/346-16). The Declaration of Helsinki was followed. *Informed consent*: according to the Swedish Data Protection Authority, the handling of data generated within the framework of quality registers represents an exception from the general rule requiring written informed consent from the patients. Furthermore, the Personal Data Act (Swedish law #1998:204, issued 29 April 1998) allows data from medical charts to be collected for clinical purposes and quality control without written informed consent.

### Variables

#### Variables used for the development of the modified Rankin Scale-Riksstroke (mRS-RS) algorithm

The reference variable mRS was derived from Väststroke's three-month follow-up data. The mRS data were collected by experienced physicians or nurses. The telephone interview guidelines described by Bruno et al.^8,12^ were followed. The mRS is used to determine a patient’s level of functional disability after stroke; it uses a 7-level ordinal scale, with 0 = "no symptoms" and 6 = "death"^5^.

Index variables were derived from Riksstroke's three-month follow-up data*.* Seven self-reported questions were selected by health care professionals based on clinical reasoning for the mapping procedure. The questions were as follows: 1. "Are you today dependent on a family member/next-of-kin for help/support?" 2. "Where are you staying now?" 3. "How is your mobility now?" 4. "Do you receive help from anybody to go to the toilet?" 5. "Do you need help getting dressed and undressed?" 6. "Are you still having problems after your stroke?" 7. "Have you been able to return to the life and activities you had before you had a stroke?" (Supplementary Table S1).

#### Variables used for describing the study sample

From Väststroke: the stroke severity at admission to the hospital was assessed with the National Institutes of Health Stroke Scale (NIHSS)^13^. Cognitive function during the hospital stay was assessed with the Montreal Cognitive Assessment (MoCA)^14^.

From Riksstroke: the patients’ sex, age, risk factors for stroke, activity status before stroke, reperfusion treatment, length of hospital stay and discharge destination were retrieved. The stroke type was classified according to the ICD-10 codes: no traumatic intracerebral haemorrhage (I61), cerebral infarction (I63) and unspecified stroke (I64). The level of consciousness upon arrival at the hospital was rated with the Reaction Level Scale (RLS). The RLS scores ranged from 1 to 8, where "1" meant fully awake/alert^15^.

### Statistical analysis

The study sample features are described as the means (SD), medians (range), counts (%) and quartiles (Q_1_–Q_3_). Drop-out analyses were performed with Pearson's χ^2^ test for nominal variables and the Mann–Whitney *U* test for ordinal and continuous variables with skewed distributions.

#### Development of the modified Rankin Scale-Riksstroke (mRS-RS) algorithm

##### Prework and mapping

A multidisciplinary group of health care professionals (three authors: T.A., M.R., K.S.S.) with 8–30 y of experience in stroke rehabilitation (research as well as clinical work) has performed the manual mapping of the Riksstroke's questions with the mRS grades. Seven self-reported questions from Riksstroke were identified as possible candidates for the mRS-RS algorithm. Furthermore, the answer categories from Riksstroke’s questions were matched with mRS grades, individually by each health care professional. The answer choices “Do not know” and "Have no relatives/friends or have no contact with relatives/friends" were excluded from the analyses.

The results of manual mapping were summarized in SPSS. The overall agreement between the mRS-RS and mRS as well as the agreement in individual grades were assessed with Cohen's kappa. Since the mRS is an ordinal scale, the Fleiss-Cohen type of quadratic weights was calculated (K_w_). The level of agreement was interpreted as slight (K_w_ ≤ 0.20) , fair (K_w_ = 0.21–0.40), moderate (K_w_ = 0.41–0.60), substantial (K_w_ = 0.61–0.80) and almost perfect (≥ 0.81)^16^. The results are described with a cross table, the K_w_ values, p-values and 95% CIs.

#### Decision trees

The reference variable mRS (Väststroke) was ordinal. There were both ordinal and nominal index variables, which were the seven questions from Riksstroke. Thus, the nonparametric method was used for building the decision trees. The dataset was divided into training (80%) and test sets (20%). Several measurements were used to evaluate the performance of the decision trees. The classification accuracy was used to identify the overall rate of correctly classified patients. Classification accuracy was further compared with the no information rate to determine the usability of the model. The quadratic K_w_ was used to study the agreement between the true and predicted values of the mRS. For each classification tree, confusion matrices were created.

Three individual decision trees were built:

- Tree 1. For the validation of the mRS-RS algorithm that was developed based on manual mapping, we used the mRS with grades 0–2, 3, 4 and 5 as a target variable.
- Tree 2. To distinguish mRS grades 0, 1 and 2 from each other, the full-scale mRS was used as a target variable.
- Tree 3. Due to clinical reasoning and manual mapping issues, the target variable was the mRS with grades 0–1, 2–3 and 4–5, which indicated no disability, moderate disability and severe disability, respectively.

Descriptive statistical analyses of the study sample and agreement analyses were performed in SPSS Statistics 26.0 (IBM SPSS Statistics for Windows, Armonk, NY: IBM Corp). Decision trees were built using the conditional inference trees (ctree) approach from the toolkit for recursive partitioning (partykit) package^17,18^ (R, version 3.6.2). All tests were two-sided and conducted at the 5% significance level.

## Results

### The study sample, baseline demographics and clinical characteristics

**Figure 1:**
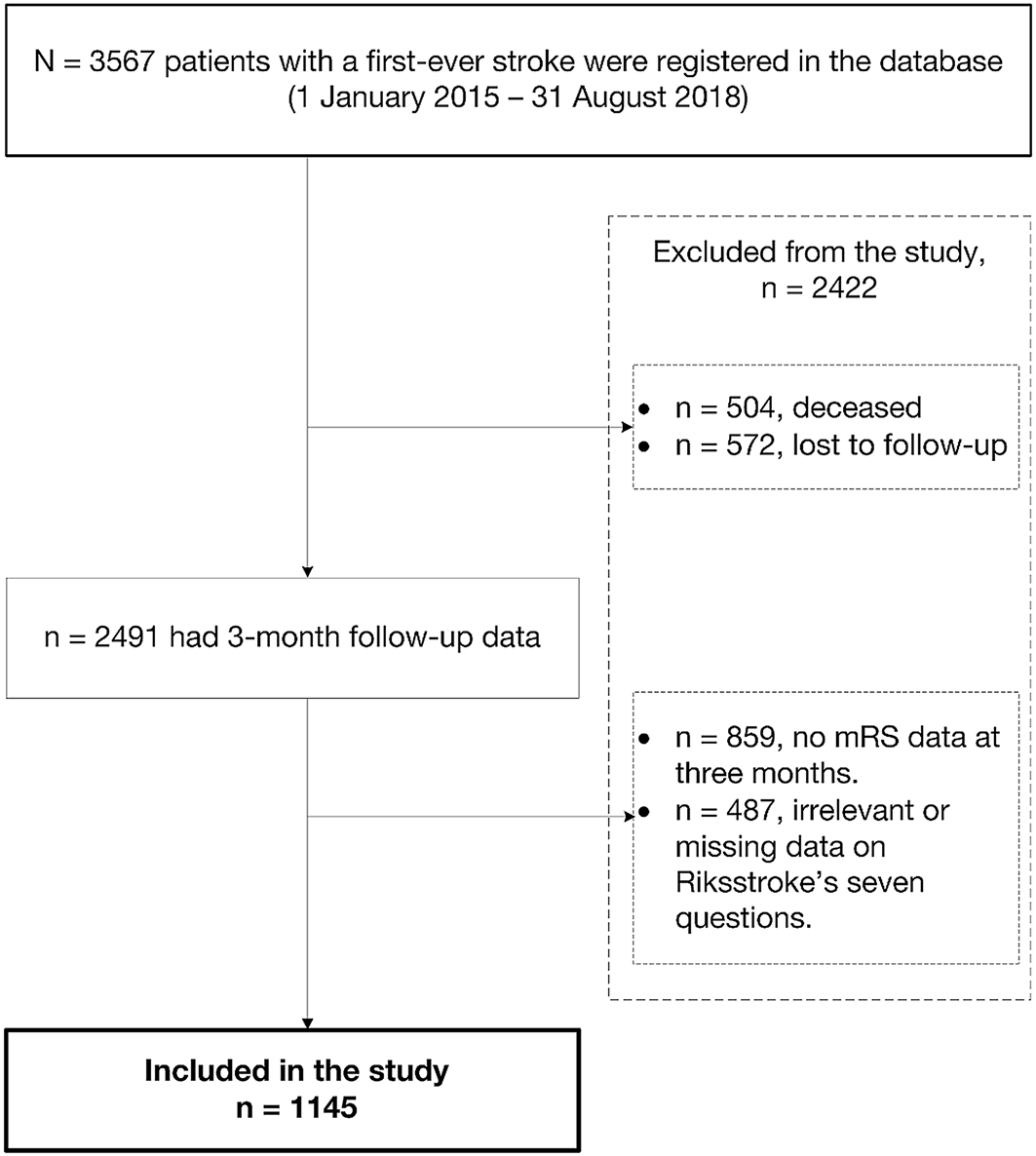
Study flowchart (mRS-modified Rankin Scale).

In total, 1145 of 3567 patients with a first-ever stroke met the inclusion criteria (Fig. 1). Patients (n = 2245) were excluded either due to death or missing data at 3 months. Compared with the included patients, the excluded patients were more male (*p* < 0.05), had more severe stroke (*p* < 0.001) and an older age (*p* < 0.001).

The mean (SD) age of the patients was 71 y (14.3 y), and 54% were male. Before the stroke, the majority of the patients lived in their own homes without community services (87%). They were independent in terms of mobility (92%), toilet visits (97%) and getting dressed (96%). At the onset of stroke, 79% of patients had a mild stroke (NIHSS ≤ 5p), 89% had cerebral infarction, and 17% of the patients with infarction had received reperfusion treatment. Furthermore, 55% of the patients showed impaired cognition (≤ 25p), as assessed with the MoCA, during the hospital stay. The majority of the patients (72%) were discharged to their own homes (Table 1). Three months after the stroke, 21% of the patients had no symptoms (mRS = 0), 86% of the patients were living in their own homes with or without community services, but only 29% had been able to return to the lifestyle and the activities they had performed before the stroke (Table 1).

**Table 1:** Baseline demographics and clinical characteristics of the study sample (n = 1145).

Baseline characteristics	
Female sex, n (%)	523 (46%)
Age in years, mean (SD*/range)	71 (14.3/19–100)
Lives in own home with/without community services, n (%)	1105 (96%)
Lives in community facility or other, n (%)	40 (4%)
Lives alone, yes/no, n (%)	501 (44%)/633 (56%)
Needed help prior to the stroke, yes/no, n (%)	134 (14%)/977 (84%)
Diabetes, yes, n (%)	187 (16%)
A history of TIA^†^, yes, n (%)	69 (6%)
Smoking, yes, n (%)	130 (13%)

Stroke-related features	
**Stroke diagnosis, n (%)**	
I61 Cerebral haemorrhage	127 (11%)
I63 Cerebral infarctions	1016 (89%)
I64 Acute cerebrovascular diseases, not specified	2 (0.2%)
Reperfusion, n (%)	183 (17%)
NIHSS^§^, median (Q_1_–Q_3_)^| |^/(range)	1 (0–5)/(0–26)
MoCA^#^, median (Q_1_–Q_3_)/(range)	25 (22–27)/(5–30)
Length of hospital stay in stroke units, days, median (Q_1_–Q_3_)/(range)	7 (4–16)/(0–100)
**Discharge destination, n (%)**	
Own home	824 (72%)
Community facility	174 (15%)
Another acute clinic	13 (1%)
Geriatric/rehab unit	132 (11%)
Other stroke units	2 (0.2%)

Functional outcome 3 months after stroke (modified Rankin Scale), n (%)	
No symptoms at all	242 (21%)
No significant disability despite symptoms	314 (27%)
Slight disability, unable to carry out all previous activities	264 (23%)
Moderate disability, requiring some help, able to walk without assistance	178 (15%)
Moderately severe disability, unable to walk without assistance	85 (7%)
Severe disability, bedridden	62 (5%)

The sum may be different because of the missing values. Abbreviations: *SD standard deviation, ^†^*TIA* a Transient Ischemic Attack*,* ^‡^*RLS* the Reaction Level Scale (the range 1–8, where 1 means fully awake), ^§^*NIHSS* the National Institute of Health Stroke Scale (the scores range from 0–42 points, a lower score indicates a less severe neurological status), ^| |^ (Q_1_—Q_3_)—the first quartile–the third quartile, ^#^MoCA—the Montreal Cognitive Assessment (the scores range from 0–30 points, a low score indicates more severe cognitive deficits).

Variables with missing data n (%), presented in alphabetical order: diabetes 1 (≤ 1%), level of consciousness upon arrival at the hospital 17 (≤ 1%), lives alone 11 (≤ 1%), MoCA score 609 (53%), needed help prior to stroke 14 (≤ 1%), NIHSS 238 (21%), a history of TIA 8 (≤ 1%), reperfusion 92 (8%), smoking 116 (10%).

### Manual mapping-first transformation algorithm of mRS-RS

The results of the new manual mapping procedure were combined with a transformation algorithm that was previously developed by Eriksson et al.^9^, leading to the first version of the mRS-RS (Table 2). The distribution density of the mRS grades from Väststroke and Riksstroke's 7 questions is presented in Supplementary Figure S1. The manual mapping results could not be used to distinguish between mRS grades 0, 1 and 2. Thus, this aim could not be fulfilled.

**Table 2:** The manual mapping results.

mRS grades	The answer choices to the self-reported questions from Riksstroke*	Answer codes as defined in Supplementary Table S1
0–2	Q1 Not or partly dependent on support or assistance from relatives/friends *OR*	3 or 4
	Q6 All problems have completely gone *OR*	1 or 2
	Q7 Can return to the life and activities I had prior to stroke	1 or 2
	Q2 Live in my own home, without community cervices	1
	Q3 Can get around both indoors and outdoors without the help of another person	1
	Q4 Can manage to visit the toilet by myself	1
	Q5 Can manage to get dressed and undressed by myself	1
3	Q1 Completely dependent on a next of kin for help/support *OR*	2
	Q2 Live in own home with community support *OR*	2
	Q3 Can move around without help at least indoors	1 or 2
	Q6 All problems have completely gone	2
4	Q3 Cannot move around without help indoors *OR*	3
	Q4 Need help to go to the toilet *OR*	2
	Q5 Need help to get dressed and undressed	2
	Q6 All problems are completely resolved	2
	Q7 Cannot return to the life and activities I had prior to stroke	3
5	Q2 Not living in my own home	3
	Q3 Need another person’s help to move	3
	Q4 Need help to go to the toilet	2
	Q5 Need help to get dressed and undressed	2

The translation algorithm used to transform the self-reported questions from Riksstroke into modified Rankin Scale (mRS) grades.

*The answers of the multiple-choice questions are given as stated in Riksstroke’s follow-up questionnaire.

To develop a new transformation algorithm, mRS grades 0, 1 and 2 were merged (Table 2). The new algorithm achieved almost perfect agreement K_w_ = 0.81 (*p* < 0.001, 95% CI 0.77–0.85), Table 3. With the new transformation algorithm, 78% of the patients were correctly classified, 6% of the patients were given lower mRS grades than the reported grades, and 15% were given higher mRS grades (Table 3, section a). The number of correctly classified patients for each mRS grade as well as agreement on the individual mRS grades are presented in Supplementary Figure S2.

**Table 3:**
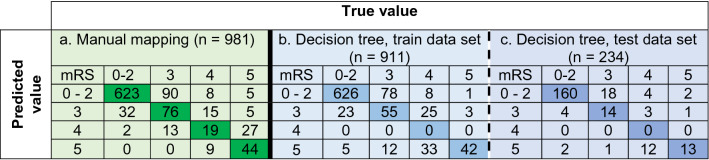
The confusion matrices of the manual mapping algorithm and the decision tree for the modified Rankin Scale grades 0—2, 3, 4 and 5.

The results are presented as the number of patients.

n = 981 patients due to characteristics of the manual translation algorithm comprising OR and AND conditions.

### Decision trees

With the decision tree in which the mRS grades 0, 1 and 2 were merged, 80% of the patients were correctly classified. The level of agreement was substantial, with K_w_ = 0.66 and 0.67 for the training and testing sets, respectively (Table 4). The proportions of the patients that were correctly classified were similar for the decision tree (the training and testing sets) and manual mapping procedures; however, the decision tree could not be used to classify the patients with a mRS grade 4, which was a major difference between the two procedures (Table 3).

**Table 4:**
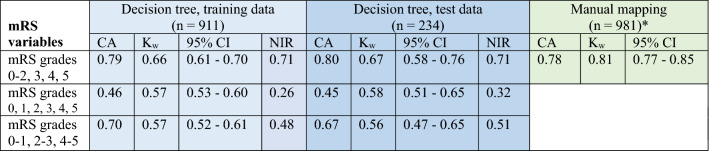
Summary statistics of the three decision trees based on different versions of the modified Rankin Scale (Väststroke) variables.

CA—classification accuracy, the rate of correctly classified patients, K_w_—quadratic weighted kappa, 95% CI—95% confidence interval for K_w_, NIR—no information rate .

*n = 981 patients due to characteristics of the manual translation algorithm comprising OR and AND conditions.

The decision tree that was used to distinguish mRS grades 0, 1 and 2 yielded correct classifications for only 46% of the patients (Supplementary Table S2—a). The overall K_w_ indicated moderate agreement (0.58) (Table 4). With the decision tree in which mRS grades 0–1, 2–3 and 4–5 were included as a target variable, 70% of the patients in the training data and 67% of the patients in the testing data were correctly classified (Supplementary Table S2—b). The K_w_ values indicated moderate agreement, as they were 0.57 and 0.56 for the test and training datasets, respectively (Table 4).

**Figure 2:**
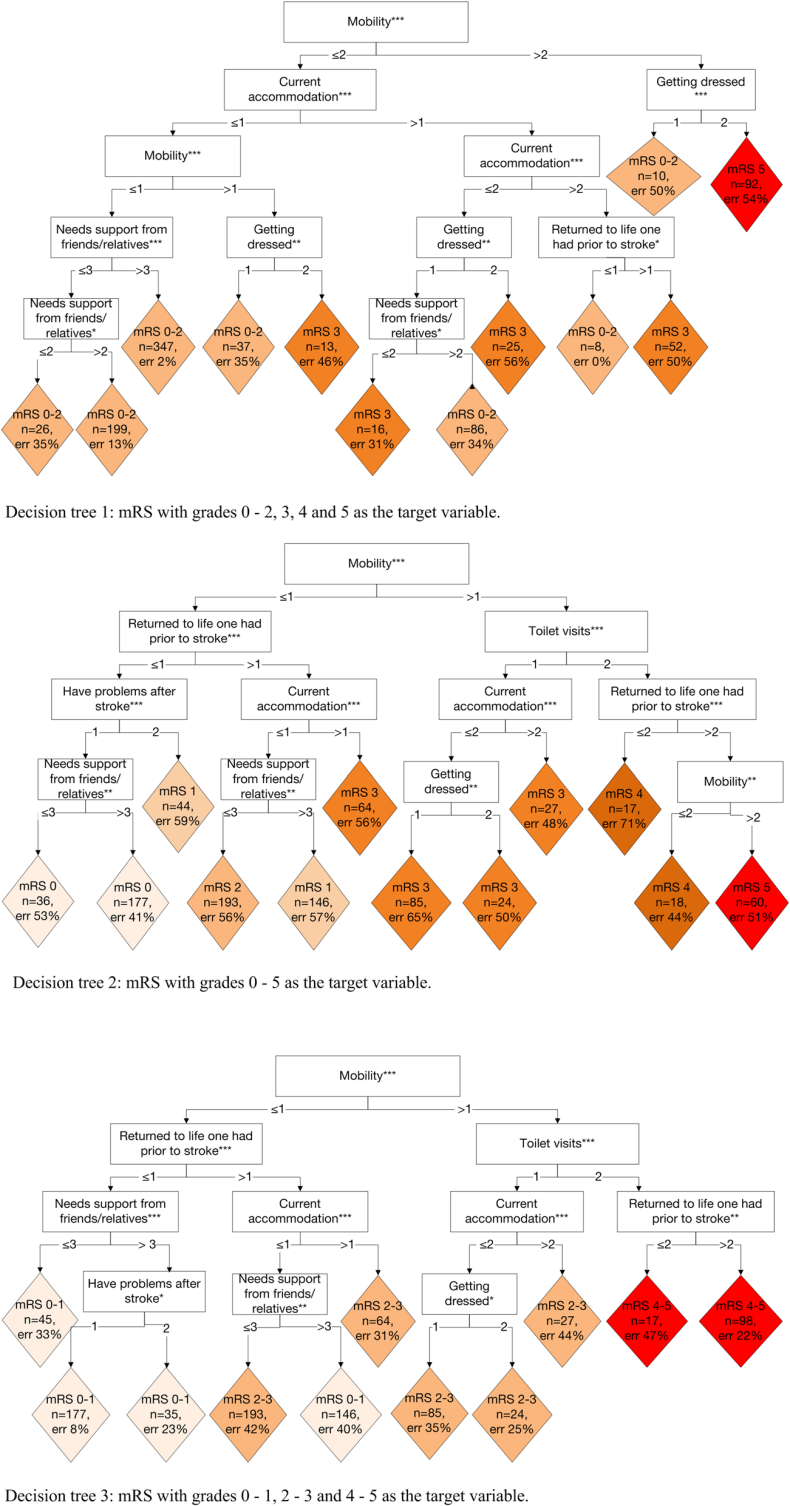
Graphical representation of the decision trees. The classification of Riksstroke’s seven questions into the modified Rankin Scale (mRS). The different colours indicate various mRS grades. *Note* ***p < 0.001; **p < 0.01; *p < 0.05.

The decision trees are presented in Fig. 2.

## Discussion

This study demonstrates a methodological approach for transforming self-reported functional outcomes from a stroke register into the mRS. Manual mapping and a supervised machine learning method, namely, decision trees, were used. The mRS grades 0, 1 and 2 could not be distinguished from each other with either of these methods; therefore, these grades were merged. Substantial classification accuracy and almost perfect agreement for manual mapping were obtained as the result of the implementation of both methodological approaches. However, the patients with mRS grade 4 could not be classified with the decision trees. Therefore, the results of the manual transformation algorithm can be used for comparative studies based on Riksstroke data, where the mRS score is an outcome variable.

One of the aims of this study was to distinguish mRS grades 0, 1 and 2. The aim was challenging, although the mapping was conducted by experienced health care professionals in stroke rehabilitation (T.A., M.R., K.S.S.). The aim could not be fulfilled after applying a machine learning method for classification purposes. There are several explanations for this result. First, although Riksstroke's questions included in the algorithm have been validated, they lack specificity in the wording. Furthermore, multiple choice questions from Riksstroke register are binary or ordinal and grouped into three categories. This limitation in the response options can lead to difficulty in distinguishing "no symptoms", "no significant disability" and "slight disability". Second, the limited accuracy of self-reported information should be considered in interpreting the results of the study. Motivational and cognitive processes of the patients can bias their responses to the self-reported questions. Third, the data on the mRS (Väststroke) were obtained by several research nurses using the telephone interview guideline^8^. Although the interview guideline has shown substantial interrater agreement^8,12^, it showed a lower level of agreement between some grades of mRS.

The other aim of the study was to create a new transformation algorithm based on the Riksstroke questions. Although mRS grades 0, 1 and 2 were merged, misclassification could not be avoided; the results were overestimated for 15% of the patients and underestimated for 6%. The current study results are relatively similar to the classification characteristics of the first transformation algorithm developed by Eriksson et al.^9^ In that study, the results were overestimated for slightly fewer patients (14.2%) underestimated for more patients (9.5%). Furthermore, the proportion of correctly classified patients was substantial in this study, which was in agreement with the results of Eriksson et al.^9^ However, Eriksson et al.^9^ reported a lower K_w_ compared with that in our study. When a machine learning method was applied for the sensitive analyses, similar levels of classification accuracy were achieved for the training and testing datasets, but mRS grade 4 could not be classified. In conclusion, it is suggested that the manual algorithm for mRS-RS, where the mRS grades are coded as 0–2, 3, 4 and 5, can be useful for the classification of functional disability.

In this study, it was challenging to distinguish mRS grades 2 from 3 and 4 from 5. The same issue was mentioned by Eriksson et al.^9^ The clinical model was created by merging mRS grades 0–1 (no functional disability/functional independence), 2–3 (moderate functional disability) and 4–5 (severe functional disability). Choosing mRS grades 0–1 for functional independence was suggested in different stroke trials ^5^ and linked with independence in everyday life as measured by the Barthel Index^19^. The decision tree model showed substantial classification accuracy and moderate agreement. Implementing this model could avoid misclassification of the patients, especially towards better outcomes that can have extensive consequences for patients' everyday lives, because of the risk that they will not receive the necessary care and rehabilitation.

This study has several strengths and limitations. Cohen's kappa is a robust statistical method feasible for studying interrater agreement^20,21^, but there are several uncertainties with kappa. Gisev et al.^21^ argue that based on the formula of the kappa, it can be difficult to achieve perfect agreement; moreover, low kappa does not always correspond to low agreement^21,22^. Hence, we have chosen to also present the classification accuracy (%). Weighted kappa represents an extension of Cohen's kappa, and it is useful for rating items with more than two categories^21,23^, studying the agreement between major categories and identifying how they differ from each other^22^. However, the K_w_ coefficients can differ by the number of categories, which makes it difficult to compare the coefficients with each other^24,25^. Furthermore, interrater agreement bias, as well as disagreement in the classification items, are difficult to avoid^26^. These issues were not present in the Eriksson et al.^9^ study, where one experienced nurse gathered the data on the mRS as well as Riksstroke's 3-month follow-up questionnaire.

This study was restricted to first strokes, and the results may differ in recurrent strokes. From a patient perspective, there can be a big difference from having no symptoms at all from a stroke (mRS 0) and having a stroke that leaves you dependent on others for certain activities (mRS 2). By combining these values, we have probably missed important information on patient recovery. The use of the full ordinal scale would be more efficient, however, the results of the manual mapping of the Riksstroke questions to mRS outcome of good (mRS grades 0–2) and poor outcome (mRS grades 3, 4 and 5) is still useful when the aim is to classify patients.

Supervised machine learning methods can be used in register-based studies^2^. By applying the decision tree method, a more sensitive method of classification was expected but only partly achieved. It is possible that the data were not balanced. Several physicians and research nurses collected the mRS data over 31 months. The physicians/nurses did not undergo training for calibration, and the interrater agreement between the nurses/physicians could not be assessed because of the high rate of employee turnover in the stroke units. Furthermore, decision trees tend to have high accuracy because of overfitting^27^. This problem was addressed by introducing the rule of minimal split at n = 50.

## Conclusions

To the best of our knowledge, this is the first study in which a combination of manual mapping and a machine learning method was applied to transform register-based self-reported functional outcomes to a standardized assessment instrument by using the data from two stroke registers. The method presented in this study can be useful for other studies with similar aims. The new transformation algorithm developed based on manual mapping might be useful for research using Riksstroke data. However, before transferring the results to other cohorts, external validation is recommended.

## Supplementary information

Supplementary information.

## Data Availability

According to the Swedish regulations shown in https://etikprovning.se/for-forskare/ansvar/, the complete dataset cannot be made publicly available for ethical and legal reasons. Researchers can request access to the data by emailing the principal investigator at ks.sunnerhagen@neuro.gu.se.
