# Supplementary material for: Transforming self-reported outcomes from a stroke register to the modified Rankin Scale: a cross-sectional, explorative study
Source: Sci Rep. 2020 Oct 14;10:17215. doi: 10.1038/s41598-020-73082-4 (PMC7560748; doi:10.1038/s41598-020-73082-4)
Supplement: Supplementary file 1 — Supplementary information. [file 41598_2020_73082_MOESM1_ESM.docx]

SUPPLEMENTAL MATERIAL

**Transforming self-reported outcomes from a stroke register to the modified Rankin Scale: A cross-sectional, explorative study.**

**Tamar Abzhandadze, MS^1,2^**, Malin Reinholdsson, MS^1,2^, Annie Palstam, PhD^1^, Marie Eriksson, PhD^3^, Katharina S. Sunnerhagen, MD, PhD^1^

^1^ Institute of Neuroscience and Physiology, Rehabilitation medicine, University of Gothenburg, Gothenburg, Sweden. Per Dubbsgatan 14, fl. 3, 413 45 Gothenburg.

^2^Department of Occupational Therapy and Physiotherapy, Sahlgrenska University Hospital, Gothenburg, Sweden

^3^Department of Statistics, USBE, Umeå University, Umeå, Sweden

[tamar.abzhandadze@gu.se](mailto:tamar.abzhandadze@vgregion.se)

**Supplemental tables**

Supplementary Table S1**.** Description of self-report questions from Riksstroke used for the mRS algorithm [the answer choices in square brackets were not analyzed].

| **Riksstroke's questions*** | **Multipel choice answers*** |
| --- | --- |
| Q1. Are you currently dependent on support or assistance from relatives/friends? | [1. Have no relatives/friends or have no contact with relatives/friends]  2 = Yes, completely dependent  3 = Yes, partly dependent  4 = No, not at all  [8. Do not know] |
| Q2. Where are you living currently? | 1= Live in my own home, without community support. (Community support does not refer to home nursing or advanced home nursing)  2 = Live in my own home, with community support. (Community support does not refer to home nursing or advanced home nursing)  3 = Does not live in own home (lives in the community facility, emergency hospital, geriatric/rehabilitation clinic or other institution). |
| Q3. How is your mobility now? | 1 = I can get around both indoors and outdoors without the help of another person  2 = I can get around indoors but not outdoors without the help of another person  3 = I get help from someone else to move around both indoors and outdoors |
| Q4. Do you need help from someone to visit the toilet? | 1 = I can manage to visit the toilet by myself  2 = I need help to visit the toilet |
| Q5. Do you need help getting dressed and undressed? | 1 = I can manage to get dressed and undressed by myself  2 = I need help getting dressed and undressed |
| Q6^†^. Are you still having problems after your stroke? | 1 = All problems have completely gone  2 = I am still having problems  [8. Do not know] |
| Q7^†^. Have you been able to return to the life and activities you had before you had the stroke? | 1 = Yes  2 = Yes, but not quite like before  3 = No  [8. Do not know] |

*The questions and the answers of the multiple choice questions are given as stated in Riksstroke’s follow-up questionnaire. ^†^ Variables were not included in the previous algorithm developed by Eriksson et al.^1^

Supplementary Table S2**.** Confusion matrices of the three decision trees. The results of the training and testing datasets are presented. The diagonal line shows the correctly classified cases (n corresponding to number of the patients).

a. Target variable - the modified Rankin Scale with grades 0, 1, 2, 3, 4 and 5.

|  | **True value** | | | | | | | | | | | | | |
| --- | --- | --- | --- | --- | --- | --- | --- | --- | --- | --- | --- | --- | --- | --- |
| **Predicted value** | Training data set (n = 911) | | | | | | | Testing data set (n=234) | | | | | | |
|  | **mRS** | **0** | **1** | **2** | **3** | **4** | **5** | **mRS** | **0** | **1** | **2** | **3** | **4** | **5** |
|  | **0** | 121 | 70 | 16 | 5 | 1 | 0 | **0** | 27 | 14 | 3 | 0 | 1 | 0 |
|  | **1** | 35 | 81 | 66 | 8 | 0 | 0 | **1** | 7 | 26 | 14 | 0 | 0 | 0 |
|  | **2** | 24 | 56 | 84 | 28 | 1 | 0 | **2** | 6 | 22 | 12 | 2 | 0 | 0 |
|  | **3** | 12 | 31 | 48 | 84 | 22 | 3 | **3** | 6 | 11 | 16 | 25 | 4 | 1 |
|  | **4** | 4 | 0 | 3 | 9 | 15 | 4 | **4** | 0 | 0 | 0 | 5 | 2 | 1 |
|  | **5** | 0 | 2 | 1 | 11 | 27 | 39 | **5** | 0 | 1 | 1 | 1 | 12 | 14 |

b. Target variable - the modified Rankin Scale with grades 0-1, 2-3 and 4-5.

|  | **True value** | | | | | | | |
| --- | --- | --- | --- | --- | --- | --- | --- | --- |
| **Predicted value** | Training data set (n = 911) | | | | Testing data set (n=234) | | | |
|  | **mRS** | **0-1** | **2–3** | **4-5** | **mRS** | **0-1** | **2-3** | **4-5** |
|  | **0-1** | 307 | 95 | 1 | **0-1** | 74 | 17 | 1 |
|  | **2-3** | 123 | 244 | 26 | **2-3** | 45 | 55 | 5 |
|  | **4-5** | 6 | 24 | 85 | **4-5** | 1 | 7 | 29 |

**Supplemental figures**

**
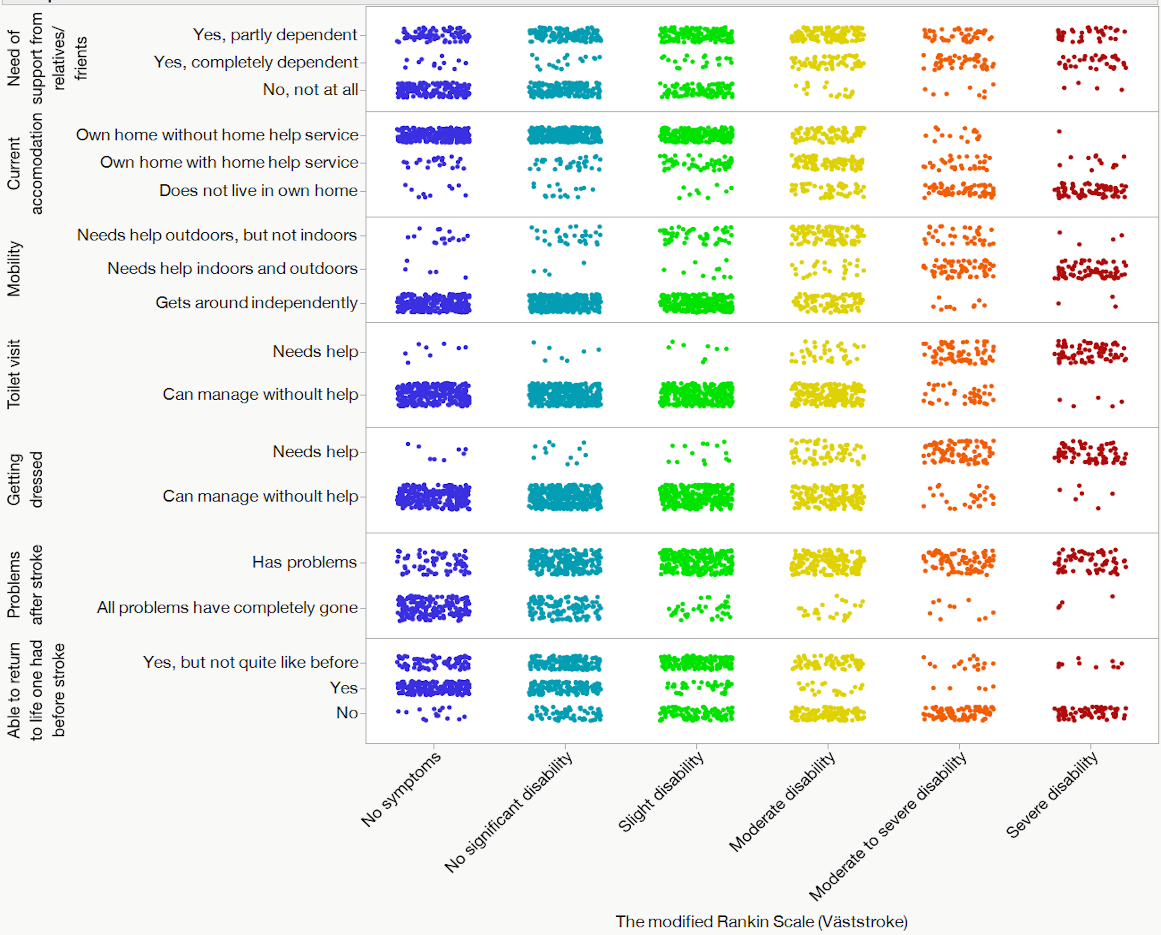
**

Supplementary Figure S1**.** A scatter plot matrix representing the distribution density between Riksstroke's 7 questions and the modified Rankin scale grades from Väststroke.


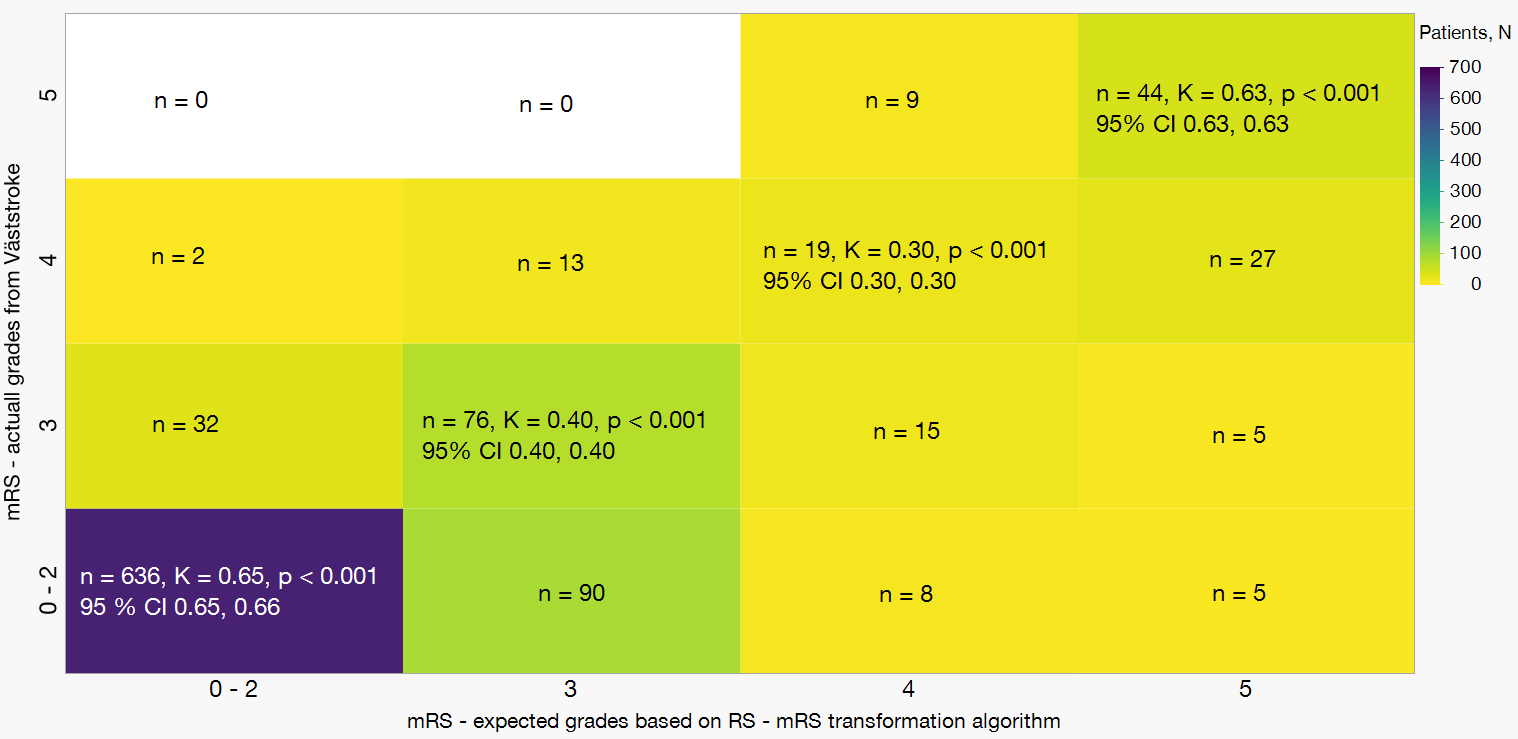
Supplementary Figure S2**.** Heatmap showing the agreement in individual grades of the modified Rankin scale (Väststroke) and mRS – RS (manual mapping). The results are presented as the number of patients in the individual cells, with the weighted kappa values (K_w_), p-values and 95% confidence intervals (95% CIs).

**Supplemental reference**

1. Eriksson M, Appelros P, Norrving B, Terént A, Stegmayr B. Assessment of functional outcome in a national quality register for acute stroke: can simple self-reported items be transformed into the modified Rankin scale? *Stroke*. **38,** 1384-1386 (2007).
